# An On-Chip Viscoelasticity Sensor for Biological Fluids

**DOI:** 10.34133/cbsystems.0006

**Published:** 2023-01-10

**Authors:** Qianbin Zhao, Sheng Yan, Boran Zhang, Kai Fan, Jun Zhang, Weihua Li

**Affiliations:** ^1^ Hebei Key Laboratory of Biomaterials and Smart Theranostics, School of Health Sciences and Biomedical Engineering, Hebei University of Technology, Tianjin 300131, China.; ^2^ Institute for Advanced Study, Shenzhen University, Shenzhen 518060, China.; ^3^ School of Electrical and Electronic Engineering, Nanyang Technological University, Singapore 639798, Singapore.; ^4^ Department of Precision Machinery and Precision Instrumentation, University of Science and Technology of China, Hefei 230026, China.; ^5^ Queensland Micro and Nanotechnology Centre, Griffith University, Brisbane, QLD 4111, Australia.; ^6^ School of Mechanical, Materials, Mechatronic and Biomedical Engineering, University of Wollongong, Wollongong, NSW 2522, Australia.

## Abstract

There are so many non-Newtonian fluids in our daily life, such as milk, blood, cytoplasm, and mucus, most of which are viscoelastic heterogeneous liquid containing cells, inorganic ion, metabolites, and hormones. In microfluidic microparticle-manipulating applications, the target particles are practically distributed within the biological fluids like blood and urine. The viscoelasticity of biological fluid is constantly ignored for simplicity especially when the fluid is substantially diluted and contains rather complex components. However, even the fluid’s ultraweak viscoelasticity actually affects the microparticle migration and may bring a completely different behavior compared with the Newtonian fluids. As a result, a robust and easy operated on-chip viscoelasticity sensor is potential and desired in many research and industrial fields, including assay sample preparation, clinical diagnostics, and on-chip sensor. In this work, we employed stable non-Newtonian fluid–polyethylene oxide (PEO) solutions with various concentrations to investigate and calibrate effects of the weak fluidic viscoelasticity on microparticle behaviors in a double-layered microfluidic channel. An analogy-based database of fluidic patterns for viscoelasticity sensing and relaxation time measurement was established. Then, we tested different biological fluids including blood plasma and fetal bovine serum and proved that they exhibited similar viscoelasticity effects to the PEO solutions with the corresponding concentration, which reached a good agreement with available results by references. The detection limitation of relaxation time can reach 1 ms. It promised a robust and integrated on-chip microfluidic viscoelasticity sensor for different biological fluids without complicated calculations.

## Introduction

It is known that non-Newtonian fluid exists everywhere around us in daily life. The rheological characteristics of biological and synthetic chemical fluids are essential in many fields, including the food processing industry, cosmetics, biomedical engineering, and clinical diagnostics [1–3]. As the examples, it has been demonstrated that the variation of blood viscoelastic characteristics is the substantial clinical symptom induced by inflammatory or vascular diseases, and the rheological change of protein would reflect in their composition, activity level, and conformation [4]. Moreover, it has been demonstrated that exploiting diluted synthetic non-Newtonian fluid like polyethylene oxide (PEO) solutions can implement microparticle manipulation through the elastic force in microfluidic technologies [5,6].

The relaxation time, *λ*, is dependent on fluid elasticity, and the magnitude of this parameter (*λ* > 0) can determine the rheological characteristics of non-Newtonian fluid whether bias to viscous fluid or elastic solid. Accordingly, the fluid relaxation time is an important parameter for viscoelasticity evaluation [7]. It is a critical factor in industrial productions, such as membrane coating, droplet formation, and agent mixing [3,8]. The standard linear viscoelastic response experiments can measure the viscous and elastic characteristics based on the variational frequency oscillation of parallel plates. However, the most challenging issue in measuring diluted biological fluid relaxation time by traditional techniques arises from the relatively small value of relaxation time, which might be down to milliseconds, such as DNA and protein solutions [6]. At the same time, the rather large sample consumption of conventional rheometer might cause the waste of rare and precious biological fluid.

Microfluidic rheometry is an emerging novel microfluidic technology to measure the rheological properties of fluids on a delicated microfluidics chip, which can overcome the shortcomings of sensitivity and operation of conventional rheometers [3,8–11]. It has a great potential to serve as the alternative to a conventional rheometer for weak rheological characteristic identification and measurement. Microfluidic rheometer leverages the dimension benefits of microfluidic technology and exhibits the advantages of small sample consumption, fast measurement, small footprint, and high integration functionality within a multifunctional system [12]. It is found that most existing microfluidic rheometers were adopted to implement the measurement of extensional viscosity and dynamic shear at different shear rates. Pan et al. [11] proposed a microfluidic rheometer to scale viscosity/shear stress under a low Reynolds number using a straight microchannel equipped with the conductive sensing membrane. Meanwhile, Choi et al. [10] introduced the comparator co-flow microchannel with reference fluids to estimate the viscosity of different protein samples under the zero shear condition.

However, the technologies mainly concentrated on measuring the shear viscosity, which was the viscosity of fluid with the shear rate function, and the relaxation time was ignored at the nascent stage. Then, some studies have focused on measuring relaxation time based on the microparticle hydraulic focusing in the microfluidic schemes. First, Zilz et al. [13] presented a microfluidic microrheometer for the measurement of relaxation time based on the elastic instability in the viscoelastic fluid. They calibrated the serpentine microchannel rheometer based on the Zimm theory and comparison with the first normal–stress difference data obtained from the shear rheometer. The proposed microfluidic rheometry enabled the accurate measurement of relaxation time of diluted viscoelastic PEO solutions as precise as 1 ms. Additionally, Koser et al. [14] presented a rheometry on a chip using the square channel with the constant pressure source, pneumatic valve, and reservoirs. It could measure the relaxation time at low strains by matching the results with the reported theoretical model. Giudice et al. [3,8] proposed a microfluidic rheometry that enabled to measure the zero shear viscosity and relaxation time at the same time, which originated from previous work about the viscoelasticity-induced migration of particle. However, the apparent disadvantage of microrheometer was the requirement of postprocessing to achieve the validated relaxation time, which could introduce human error. So far, various techniques to accurately measure relaxation time have been proposed. However, the difficulties in device operation and parameter evaluation due to the esoteric theory hinder the development and popularization of these proposed prototypes.

**Fig. 1.**
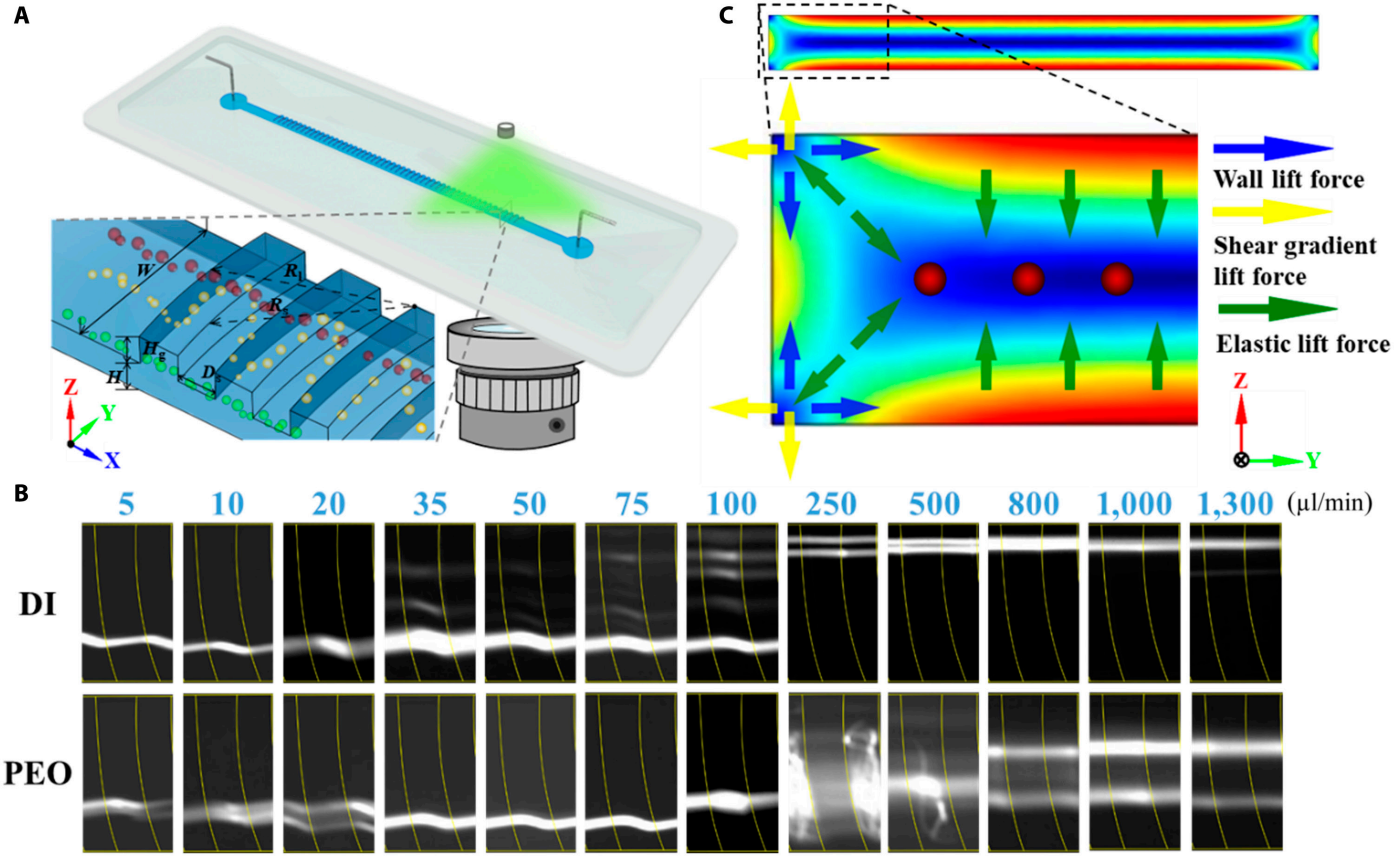
(A) Schematic figure of the 2-layer microchannel and the microparticle trajectory in non-Newtonian fluids. (B) Microparticle distribution of 9.9 μm at an outlet under flow rates of 5 to 1000 μl/min, with 25-ppm concentration of PEO. (C) Top view of the shear rate square contour (${\overset{\cdot }{\gamma }}^{2}$). The contour bar could be founded in the Supplementary Materials.

In this work, we proposed a robust, easily operating, and in situ on-chip sensor for fluid viscoelastic characteristics (Fig. 1). It employed a double-layered microchannel to identify and measure sample viscoelasticity and relaxation time. First, the effects of non-Newtonian fluids (PEO aqueous solutions) with different viscoelasticity were comprehensively investigated to establish measurement calibration. It was demonstrated that the lateral migration of 9.9-μm particles across the microchannel was extremely sensitive to the PEO concentrations (from 5 to 500 parts per million (ppm)), which was identified to be the fundamental mechanism of the viscoelasticity sensor. In microfluidic technologies, programmable particle distribution or flow pattern have been successfully exploited as the assay strategy in some biological applications, which is the working principle of the proposed viscoelasticity chip sensor [15,16]. In addition, the influences of particle size, flow rates, and molecular weight (*Mw*) of polymer on microparticle lateral migration were also investigated. Subsequently, the viscoelasticity of several common biological fluids were measured, including blood plasma, fetal bovine serum (FBS), and bovine serum albumin (BSA) solutions, and their relaxation time was estimated via analogy method. According to the experimental results, it was suggested that both FBS and blood plasma perform the viscoelastic effects in a weak but non-negligible manner. However, BSA exerted no effects on microparticle migration, which was similar to the deionized (DI) water.

## Theory

In this work, the proposed on-chip viscoelastic sensor is based on the microbead migration behavior under a synergistic inertial and viscoelastic effect. In the double-layered microchannel, multiple external forces exerted the flowing suspended microparticles. Three forces (viscoelastic forces, inertial lift, and secondary drag) dominantly influenced and modulated the bead migration at the *Re* range from ~1 to ~100. The observation of the trajectory pattern of the bead was dependent on the fluid viscoelasticity, which can be applied to the estimation of rheological property (relaxation time).

### Inertial effects

The inertia effects are usually ignored in microfluidic systems, since in most cases, the fluids appear within low Reynolds number regimes (*Re* = *ρ*_f_*UH*/*μ*, where *ρ*_f_ denotes to the fluid density, *U* denotes to the flow velocity, *μ* denotes to the dynamic viscosity, and *H* denotes to the hydraulic diameter). However, in some recent studies, it was found that the inertial effects may also affect the microsphere trajectories in some certain cases. Inertial effects were experimentally identified and were employed to manipulate suspended microparticles (namely, inertial microfluidics) [17]. Inertial migration was identified as a microfluidic phenomenon in which the randomly dispersed microparticles would gradually transfer to the positions of equilibrium in the cross section when flowing through a microchannel within a specific range of Reynolds number [18,19]. Inertial migration is dominantly determined by the wall-induced lift force and the shear gradient lift force [20]. The net inertial lift force loaded on the neutrally buoyant microspheres can be simplified as [21]:

$$F_{L}=f_{L}\rho_{f}U^{2}a^{4}/H^{2}$$ (1)

where *a* is the diameter of microsphere and *f*_L_ is the dimensonless coefficient of lift force.

In addition, the secondary flow also plays substantial roles in inertial microfluidics. In the transverse direction of the velocity-mismatched streamlines, specifically designed microchannel structures like a curved channel, obstacle, expansion, and contraction can induce a gradient of pressure [17]. Consequently, pressure-driving enclosed vortices can be generated in the cross section of the microchannel and provide the secondary flow drag force on suspended microparticles, which can be calculated by [22]:

$$F_{D}=3\mathrm{\pi a\mu}\left(v_{f}-v_{p}\right)$$ (2)

where *v*_f_ denotes to the fluid velocity and *v*_p_ denotes to the velocity of microparticles.

### Viscoelastic effects

For the non-Newtonian fluids, in addition to the inertial effects, the microparticle trajectory is also affected by the viscoelasticity of liquids. In a rectangular microchannel, the viscoelastic effects on suspended microparticles can be expressed by a dimensionless variable, named Weissenberg number (*Wi*):

$$\mathrm{Wi}=\lambda \overset{\cdot }{\gamma }=2\mathrm{\lambda U}/H$$ (3)

where *λ* is the relaxation time and *γ* is the characteristic shear rate. As the nonuniform stress differential induces an elastic lift force exerting on the microparticles, tension would be induced along the streamline by the streamwise normal stress differential (*N*_1_, *τ*_11_-*τ*_22_), and the secondary tension would be induced by the secondary normal stress differential (*N*_2_, *τ*_22_-*τ*_33_) in the cross sections as well [23–26], where *τ*_11_, *τ*_22_, and *τ*_33_ are the normal stress components in the three directions of Cartesian coordinate system. Since the intensity of the *N*_2_ is way smaller than the *N*_1_, it can be reasonably neglected and the microparticle-endured elastic force-suspended microparticles can be approximately calculated by:

$$F_{e}=C_{\mathrm{el}}a^{3}\nabla =-2C_{\mathrm{el}}a^{3}\lambda \nabla {\overset{\cdot }{\gamma }}^{2}$$ (4)

where *C*_el_ denotes to the elastic lift coefficient [27]. The elastic lift force direction is always pointing to the area of the lowest shear rate. In a high-aspect ratio channel (*w*/*h* >>1), suspended microparticles prefer to focus at the central plane, shown at the top Fig. 1C.

$$\lambda =18\lambda_{z}{\left(c/c^{*}\right)}^{0.65}$$ (5)

It was proved that inertial viscoelastic microfluidics can focus the suspended microparticles at equilibrium positions under moderate flow conditions. Furthermore, the viscoelastic and inertial effects would induce a subtle influence on the microparticle migration across the microchannel. Therefore, on the basis of measurements of the suspended microparticles trajectory, which is substantially relevant to liquid viscoelasticity, the liquid viscoelasticity may be estimated through statistic postanalysis. This is the working principle of the microfluidic viscoelasticity sensor proposed.

## Materials and Methods

### Design and fabrication of the on-chip viscoelastic sensor

The microfluidic sensor employs a polydimethylsiloxane microfluidic microchannel for the rheology sensing functionality. The double-layered microchannel with additional groove array was prepared by photolithography with 2 steps [28]. The first layer was a straight rectangular channel. Meanwhile, the second layer was an arc-shaped groove array of 70 repeating structures. The cross section of the first layer has a width of 200 μm × height of 20 μm. The aspect ratio (*H*/*W*) of which was 0.1, shown as Fig. 1A. For the second layer, the arc-shaped pattern had a larger front curvature whose radius was 650 μm (*R*_l_) and a smaller back curvature whose radius (*R*_s_) was 600 μm. The groove height (*H*_g_) was 20 μm. Meanwhile, the spacing between 2 adjacent grooves (*D*_s_) was 50 μm.

### Materials

In this experimental study, the microsphere was the fluorescent polystyrene (PS) beads at commercial level (Thermo Fisher Scientific), whose diameter was 4.8 μm [catalog no. G0500, 5% coefficient of variation (CV)], 9.9 μm (catalog no. G1000, 5% CV), and 13 μm (catalog no. 36-4, 16% CV). The fluorescent PS beads were dispersed in DI water. Tween 20 (0.1% w/v; Sigma-Aldrich) was added in the DI water to avoid aggregation of beads and adhesion onto the microchannel walls.

In addition, PEO (Sigma-Aldrich), the flexible long-chain polymer powder, was also dissolved into the sample liquid, accomplishing the homogeneous non-Newtonian fluids. The PEO concentrations were 5, 10, 25, 50, 100, 250, and 500 ppm. For each concentration, the PEO powder with *Mw* of 2,000,000 and 4,000,000 were utilized.

The experimental blood sample was donated from a healthy person, taken by anticoagulant-coated vacutainer tubes (Vacuette). The obtained blood was instantly centrifuged at 4,000 rpm for 5 min. Then, the pure-plasma supernatant was obtained. PS microbead was injected and suspended in FBS, the BSA, and the blood plasma to study the viscoelastic effects of different bioliquids. FBS is a growth supplement widely used for cell culturing, it is commonly involved in cellular sample preparations. In this study, BSA (Sigma-Aldrich, *Mw* = 66,000) was introduced into DI water to form 12 mg ml^−1^ solution. Then, the liquid was stirred overnight on a shaker before usage.

### Image acquisition and processing

A syringe pump (Legato 100, Kd Scientific, USA) was used to control the flow rate. An inverted microscope (CKX41, Olympus, Japan) equipped with a high-speed charge-coupled device camera (Optimos, Q-imaging, Australia) was used to record the fluorescent microbead trajectory, with an exposure time of 15 ms. For each scenario, 50 images were recorded at the same time and stacked in Q-Capture Pro 7 (Q-imaging, Australia), a commercial image processing software.

## Results and Discussion

### Calibration of the microfluidic viscoelastic sensor

#### i. Microbead migration in PEO solutions

**Fig. 2.**
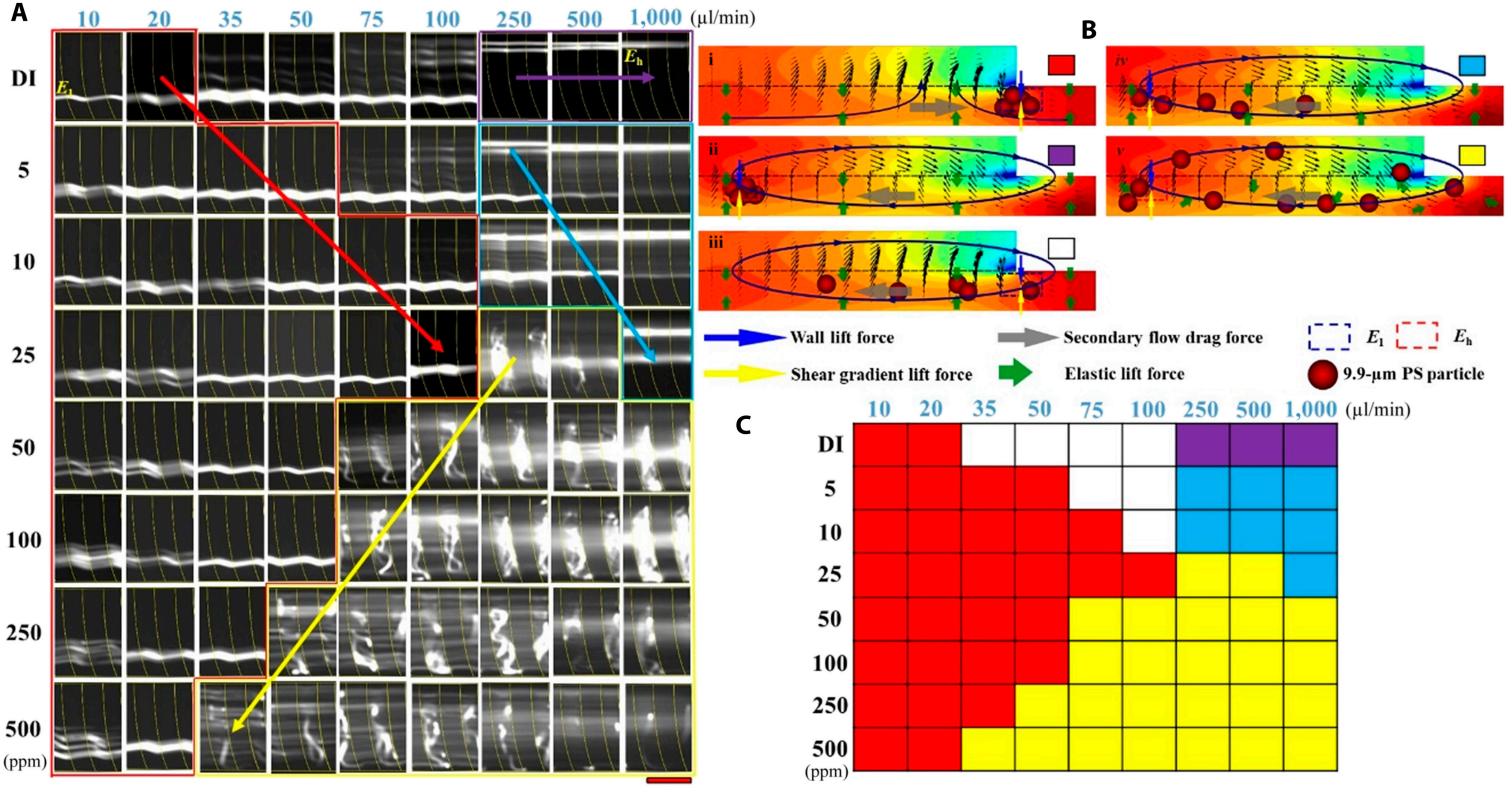
(A) Particle trajectories of 9.9-μm beads at the outlet the microchannel. The red arrow indicates the hydrophoretic focusing. The purple arrow indicates the secondary flow focusing. The weak viscoelasticity of PEO solutions deteriorated the secondary flow focusing, as indicated by the blue arrow. The green line indicates the disturbance of elastic lift force enhancement on the microbead focusing. In the yellow region, the elastic turbulence and deterioration of hydrophoretic focusing simultaneously existed. Scale bar was 100 μm. (B) Flow field distribution and bead migration at different flow conditions. i, Elastic-assisting hydrophoretic focusing (red square); ii, secondary flow focusing (purple square); iii, transitional section (white square); iv, elastic-deteriorating secondary flow focusing (blue square); v, elastic turbulence region (yellow square). (C) Microbead migration mappings. The color of units represented the corresponding microbead focusing regimes.

In the microchannel, the viscoelasticity characteristics of fluid could modulate the bead migration trajectory, which is leveraged to identify and estimate the viscoelastic magnitude of the suspension base medium. First, the influences of viscoelastic PEO solutions on the microbead trajectory were totally investigated within the 2-layer microchannels. The PS beads were focused in the ultradiluted PEO solutions with the concentration ranging from 5 to 50 ppm where the weak effects of liquid viscoelasticity was normally neglected in conventional microfluidic applications [5]. Microbeads (9.9 μm) were injected and mixed in the prepared PEO (*Mw* = 2,000,000) with a solution of 4 × 10^5^ counts ml^−1^. The PEO concentrations were controlled as 0, 5, 10, 25, 50, 100, 250, and 500 ppm. The flow rates varied from 10 to 1,000 μl min^−1^. According to captured fluorescent trajectories of particles (Fig. 2A) and the normalized intensity profiles (Fig. S1), it can be demonstrated that the final distribution would be substantially varied in different concentrated PEO solutions. Microbead trajectory within DI water was also conducted as the control experiment, where the microbeads were focused close to the left sidewall at low flow rates (10 ~ 20 μl min^−1^) but migrated to the right sidewall at high flow rates (500 ~ 1,000 μl min^−1^). In the regimes of focusing, the equilibrium position *E*_l_ at low flow rates was determined by the structure-induced hydraulic pressure fields, namely, “hydrophoresis.” Meanwhile, the equilibrium position at high flow rates *E*_h_ was determined by a stronger geometry-induced secondary flow [29,30]. As the PEO concentration increases from 0 to 25 ppm, microbeads at low flow rate were progressively focused onto a single streamline. This tendency was illustrated by the red arrow in Fig. 2A. On the contrary, the blue arrow showed that the focusing performance at high flow rates was yet getting worse with the increase of PEO concentration. Even though a small portion of 9.9-μm microbeads was still manipulated by *E*_h_, the majority of microbeads were observed to escape from the equilibrium position and randomly disperse across the microchannel.

Specifically, 9.9-μm microbeads showed the best hydrophoretic focusing in the flow rate range of 10 to 100 μl min^−1^, in the condition of 25-ppm PEO solutions. At the flow rate of 100 μl min^−1^ (Fig. S2A), the equilibrium position of microbeads migrated toward the centerline of the channel, indicating an instability among the inertial lift force, the secondary drag force, and the elastic lift force at a critical condition. Meanwhile, when the flow rate increased to 250 μl min^−1^, microvortices were generated by the grooved microstructures, which were identified as the elastic turbulence. Additionally, the higher concentration of PEO solutions (≥25 ppm) squeezed hydrophoretic focusing. For example, the upper limit of particle focusing dropped to 20 μl min^−1^ for 500 ppm of PEO solutions.

Different from our common knowledge on the negligible viscoelastic effect, PEO solutions with ultralow concentration could affect the migration of microbeads by the ultraweak viscoelastic effect. The critical working flow rate was dependent to the PEO concentration (e.g., 100 μl min^−1^ for 10 ppm of PEO solutions, 60 μl min^−1^ for 100 ppm of PEO solutions (Fig. S2B), and 40 μl min^−1^ for 250 ppm of PEO solutions (Fig. S2C).

In the channel with grooved structures, the mechanism of particle focusing is the resultant of inertial lift force, the secondary flow drag force, and the elastic lift force. Previously, it was proved that the geometry-induced secondary flow rotates constantly at high flow rates in the Newtonian fluid, driving microbeads to *E*_h_ [28,29]. However, at the low flow rates, the Dean flow drove microbeads to the opposite sidewall of the microchannel [29]. In this study, viscoelastic effects also affected the inertial focusing and approached complicated patterns of results, shown as Fig. 2A.

Subsequently, the complicated bead focusing patterns were divided into 5 different regimes, according to the counteraction among the 3 dominant hydraulic forces:

1. Elastic-assisting hydrophoretic focusing, *F*_e_ < *F*_D_, *F*_L_ ~ 0.

Determined by the elastic lift force, microbeads were manipulated toward the central plane of the microchannel. Hydrophoretic vortices would drive the bead to *E*_l_ more efficiently (Fig. 2B (i) and red region in Fig. 2C).

2. Secondary flow focusing, *F*_L_ > *F*_D_ >> *F*_e_.

Secondary flow drag force guides microbeads to *E*_h_ and was balanced with inertial lift force (Fig. 2B (ii) and purple color in Fig. 2C).

3. Transitional section, *F*_D_ > *F*_L_ or *F*_e_.

In this condition, particles experiencing the inertial lift force, the elastic lift force, and the secondary flow drag force were in the imbalance status (Fig. 2B (iii) and white region in Fig. 2C). It occurred within the transitional flow rate range ~20 to ~250 μl min^−1^, in which neither the elastic force nor the inertial lift force was able to balance the intensive secondary flow drag force.

4. Elastic-deteriorating secondary flow focusing, *F*_L_ + *F*_e_ < *F*_D_.

The viscoelastic force broke the balanced status of the secondary flow focusing (Fig. 2B (iv) and blue region in Fig. 2C). This condition usually occurs at the flow rate of ≥250 μl min^−1^ with a low concentration of PEO solutions (≤25 ppm).

5. Elastic turbulence.

The turbulence would arise from expansion–contraction structures of the grooved channel, and the generated turbulent vortices would trap and twist the microbeads within the grooves (Fig. 2B (v)) [31,32]. This condition usually occurs at the flow rate of ≥35 μl min^−1^ with a high concentration of PEO solutions (≥25 ppm). It was shown by the yellow region in Fig. 2C.

#### ii. Microbead migrating evolution along the channel length

**Fig. 3.**
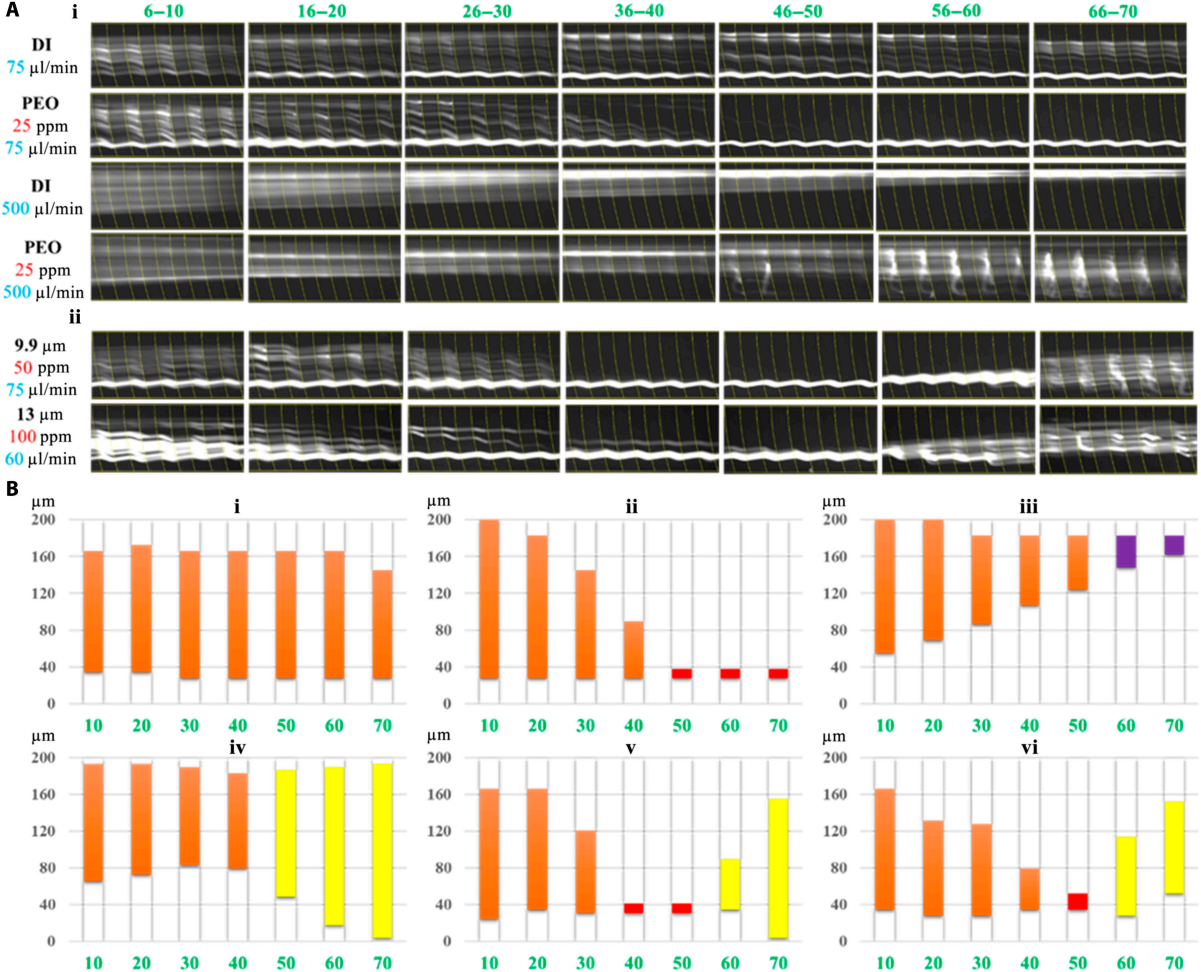
(A) The trajectories of microbeads in the grooved channel. The channel has 70 grooves, indicated by the green numbers. i. The microbeads migration in DI water and PEO solutions. ii. Intriguing microbead “dispersion–focusing–dispersion” phenomenon of 9.9- and 13-μm microbeads migrated in the microchannel. (B) Microbead distributions along microchannel according to the normalized fluorescent intensity, under different PEO concentrations and flow rates: (i) DI water, 75 μl min^−1^; (ii) 25-ppm PEO, 75 μl min^−1^; (iii) DI water, 500 μl min^−1^; (iv) 25-ppm PEO, 500 μl min^−1^; (v) 50-ppm PEO, 75 μl min^−1^; and (vi) 100-ppm PEO, 60 μl min^−1^. The colored columns represent the microbead distribution across the microchannel, and the colors suggest the focusing patterns consistent with the color mapping identified in Fig. 2C.

The migration evolution of microbeads in the grooved channel was investigated, as depicted in Fig. 3. At the flow rate of 75 μl min^−1^, the 9.9-μm microbeads were well concentrated to *E*_l_ and formed a single focusing streak after the 46th groove in the 25-ppm PEO solutions. On the contrary, they could not be focused in DI water, shown as Fig. 3B (i and ii). Nevertheless, microbeads were concentrated at *E*_h_ from the 66th groove structure in DI water at 500 μl min^−1^, while the focusing mode was deteriorated in the 25-ppm PEO solutions, shown in Fig. 3B (iii and iv).

Figure 3A (ii) and B (v and vi) shows that the 9.9-μm microbeads first gradually migrated to the left side and were well focused onto a single streamline and subsequently dispersed again in the downstream microchannel with 50 ppm of PEO at 75 μl min^−1^. In addition, the “dispersion–focusing–dispersion” phenomenon can only occur in the PEO solutions of relatively high concentrations (≥~25 ppm). It has to be noticed that the “second dispersion” was different from randomly distribution at the inlet (“first dispersion”). Different from the stable laminar microbead distribution at inlet, the “second bead dispersion” was more chaotic because of the effects of turbulent vortex in the microchannel. A similar phenomenon was also observed for the 13*-*μm microbeads. This “dispersion–focusing–dispersion” phenomenon is attributed by the viscoelastic effect.

#### iii. Effects of PEO Mw and microbead size

**Fig. 4.**
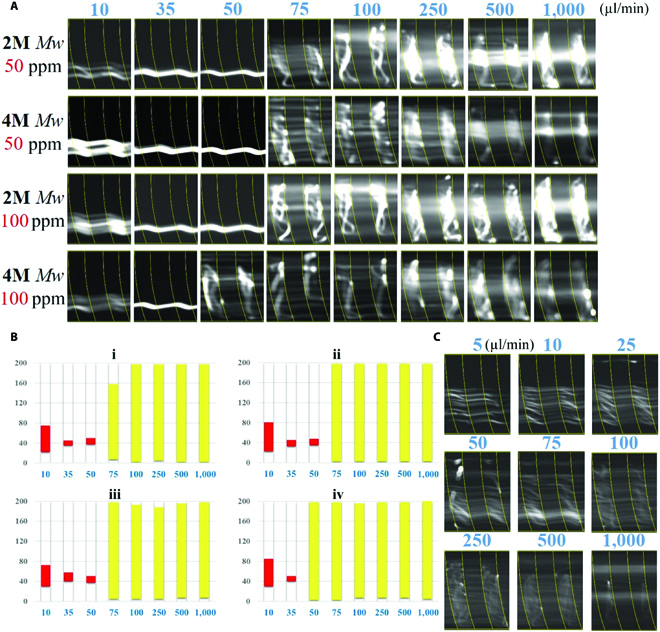
(A) The 9.9-μm particle trajectories in the PEO solutions with the *Mw* of 2M and 4M *Mw* at flow rates from 10 to 1000 μl min^−1^. (B) Particle distribution obtained from the normalized fluorescent intensity. (C) Particle trajectories of 4.8-μm microbeads in the 25-ppm PEO solutions.

In addition, we investigated the effects of the PEO *Mw* and microbead size on the particle migration, as shown in Fig. 4. With 50 ppm of PEO, the elastic turbulence was intensified by the large *Mw* such that the particles had a wider distribution at 75 μl min^−1^, shown in Fig. 4B (i and ii). With 100 ppm of PEO, the turbulence occurred at 50 μl min^−1^, compared with the condition with the smaller *Mw* PEO. This is attributed by the larger *Mw* offering a stronger viscoelastic effect with a longer relaxation time. For the particle size, a larger microbead (13 μm) showed a similar migration tendency with 9.9-μm microbeads, as shown in Fig. 3A (ii). In contrast, smaller microbeads of 4.8 μm remained in unfocusing status from 5 to 1,000 μl min^−1^, shown as Fig. 4C. The 4.8-μm particles experiencing insufficient elastic lift force and secondary drag force were unable to be focused at the equilibrium positions.

### On-chip viscoelastic measurement of biological fluids

**Fig. 5.**
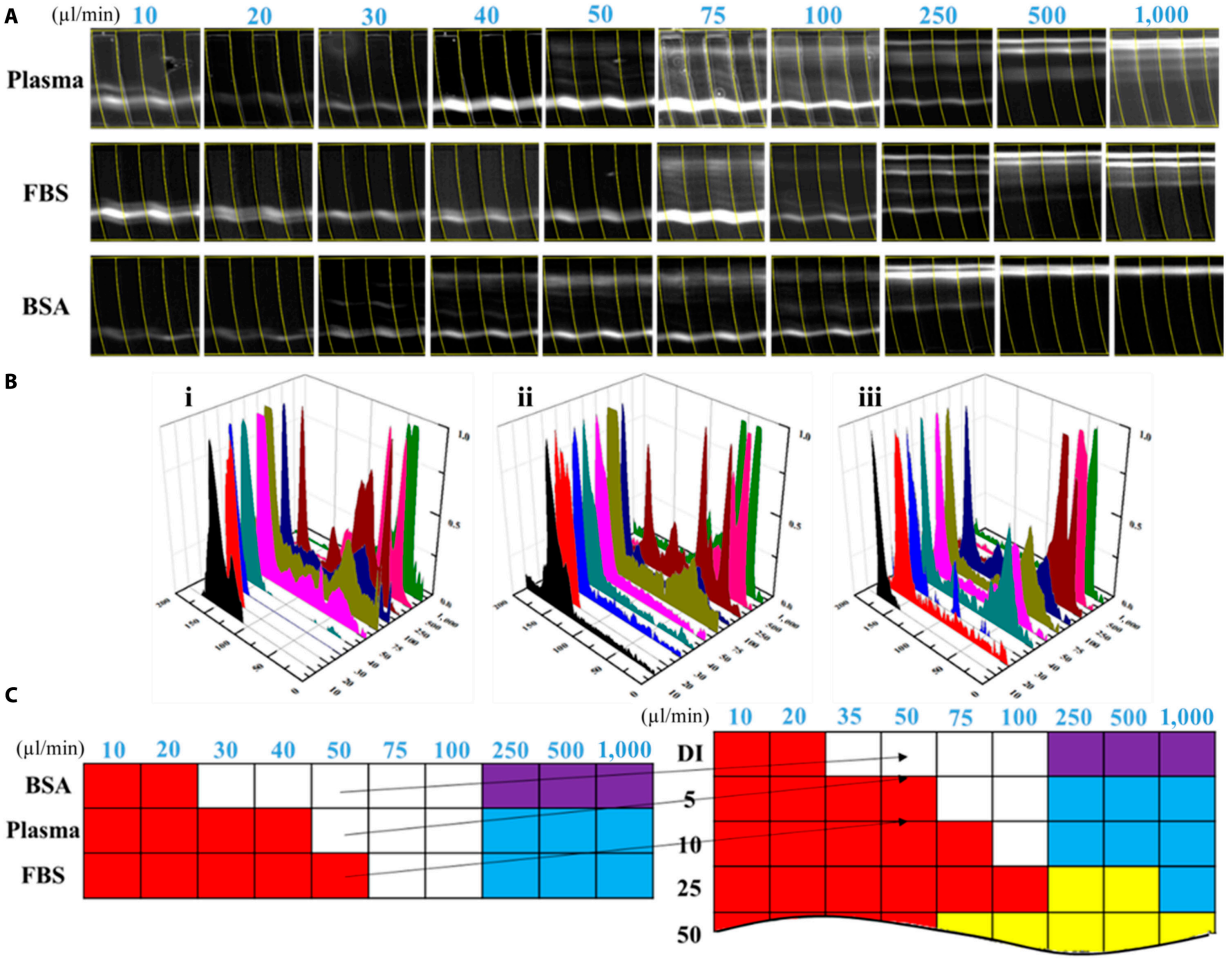
(A) Particle trajectories of 9.9-μm microbeads in blood plasma, FBS, and BSA solutions. (B) The obtained fluorescent intensity profiles indicating the microbead distribution across the microchannel at the flow rates from 10 to 1000 μl min^−1^. (C) Color mappings of the 3 biological fluids.

On the basis of the calibration of sensitivity on PEO solutions, it was demonstrated that the liquid viscoelasticity substantially affects the microbead migrating evolution along the double-layered microchannel. Reversely, this microchannel has the potential to serve as a sensing device evaluating the fluidic viscoelasticity. Many biofluids from the human body like saliva, sputum, blood, and pleural effusions are known to have viscoelasticity. In this study, 3 commonly used biofluids including blood plasma, FBS, and BSA solutions, often treated as the Newtonian fluid [33–35], were measured as representatives. PS microbeads (9.9 μm) were respectively dispersed into the plasma, FBS, and BSA solutions and then injected into the grooved channel with the flow rates ranging from 10 to 1,000 μl min^−1^. As depicted in Fig. 5, 9.9-μm microbeads could be well focused within the flow rates from 10 to 40 μl min^−1^ at *E*_l_ in the pure plasma and will be out of focus at the flow rate of 500 to 1,000 μl min^−1^. Whereas for Newtonian fluid, 9.9-μm microbeads can only be focused at *E*_l_ below 20 μl min^−1^ and at *E*_h_ when over 500 μl min^−1^. Therefore, the blood plasma has the weak viscoelasticity to influence the particle migration.

For the FBS solutions, 9.9-μm microbeads performed a better focusing at *E*_l_ from 10 to 50 μl min^−1^, while no focusing was observed at *E*_h_ at high flow rates. Therefore, it can be concluded that the FBS solution has a viscoelasticity similar to blood plasma. Moreover, BSA is the key protein in human blood plasma (more than 50% of the whole proteins in blood plasma) [36]. Microbeads (9.9 μm) were dispersed in the 10-ppm BSA solution and then injected into the microchannel. However, the migration of microbeads in BSA solutions was the same as in DI water, suggesting that BSA did not offer viscoelasticity.

In order to measure the relaxation time of biological liquids, an analogy method was further proposed for rapid estimation. On the basis of comparison between the color patterns of the biofluids and the calibrated mapping of PEO, as shown in Fig. 5C, one can effectively conclude that the plasma and FBS had the similar viscoelasticity to 5 ppm (relaxation time *λ* = 0.45 ms) and 10 ppm of PEO solutions (relaxation time *λ* = 0.7 ms), respectively. The relaxation time of various concentration PEO solutions was estimated according to the following Eq. 5:

$$\lambda =18\lambda_{z}{\left(c/c^{*}\right)}^{0.65}$$ (5)

where *λ_z_* is the Zimm relaxation time (=7.1 × 10^−4^ s) and c^*^ is the polymer overlap concentration (=858 ppm). These theoretical results are matched with previous works using the capillary breakup extensional rheometer (CaBER), in which the relaxation time of blood plasma was identified to be within 0.43 to 0.57 ms [37].

The weak viscoelasticity of biofluids like blood plasma, BSA, and FBS solutions can be negligible in the consensus of previous microfluidic research. Newtonian fluid (DI water) with applicable-sized microbeads was often utilized to explore the biological microparticles’ migration in bodily fluids, which may misguide the evaluation. The main finding of this work is that the weak viscoelasticity of biofluids cannot be neglected and can greatly affect the microparticles’ migration, and other fluidic alternatives can also be considered. Likewise, one can refer to the calibrated mapping of PEO mapping and then determine the viscoelasticity of the uncertain biofluids by observing the particle migration in the biofluids to be detected.

## Conclusions

In this study, the ultraweak viscoelasticity of liquids could greatly influence the microbead migration evolution and their distribution along a 2-layer microchannel with grooves array. According to the effects of liquids’ viscoelasticity on microparticle manipulation, an on-chip sensor was proposed for evaluating viscoelasticity of biological fluids. It was demonstrated that an imperceptible 5-ppm concentration change of dispersed PEO solutions could be clearly reflected in microbead trajectory throughout the double-layered microchannel. On the basis of the integrated high-speed camera, the sensing sensitivity could reach 1 ms. In application, 3 kinds of common biological fluids were measured and estimated using the proposed sensor and method. The results showed that both the FBS solution and the pure blood plasma had shown the viscoelastic effects on the manipulation of microbeads. Furthermore, by referring the calibrated PEO mappings, one can effectively and accurately evaluate the viscoelasticity of different biological fluids.

## Data Availability

The data used to support the findings of this study are available from the corresponding authors upon request.
