# Supplementary material for: An On-Chip Viscoelasticity Sensor for Biological Fluids
Source: Cyborg Bionic Syst. 2023 Jan 10;4:0006. doi: 10.34133/cbsystems.0006 (PMC10076049; doi:10.34133/cbsystems.0006)
Supplement: Supplementary Materials — Figs. S1 and S2 [file cbsystems.0006.f1.docx]

# An on-chip viscoelasticity sensor for biological fluids

Qianbin Zhao^a^*^#^, Sheng Yan^b^*, Boran Zhang^c#^, Kai Fan^d^, Jun Zhang^e^, Weihua Li^f^

^a^ *Hebei Key Laboratory of Biomaterials and Smart Theranostics*, *School of Health Sciences and Biomedical Engineering, Hebei University of Technology, Tianjin 300131, China*

^b^ *Institute for Advanced Study, Shenzhen University, Shenzhen 518060, China*

^c^ *School of Electrical and Electronic Engineering, Nanyang Technological University, Singapore 639798, Singapore*

^d^ *Department of Precision Machinery and Precision Instrumentation, University of Science and Technology of China, Hefei 230026, China*

^e^ *Queensland Micro and Nanotechnology Centre, Griffith University, Brisbane, QLD 4111, Australia*

^f^ *School of Mechanical, Materials, Mechatronic and Biomedical Engineering, University of Wollongong, Wollongong, NSW 2522, Australia*

*^*^ Corresponding authors:* [qz260@uowmail.edu.au](mailto:qz260@uowmail.edu.au); shengyan@szu.edu.cn

^#^ The authors contribute equally.


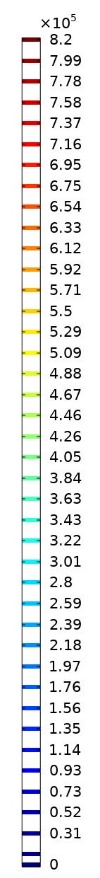


The contour bar of calculated shear rate squared (γ ̇^2), unit: 1/s^2^.

The numerical simulation of flow field was carried out using the finite element software (COMSOL Multi-physics 5.1, Burlington, MA). For the typical microfluidic system, Reynolds number was below 2000, so the steady laminar flow physics was employed in the 3D microchannel model. The boundary condition was set as “no slip” and the physical property of fluid was incompressible flow. We selected the order of finite element as P1+P1. The 3D model of the channel was meshed with free tetrahedral grid at the default finer level to ensure the accuracy of numerical results.


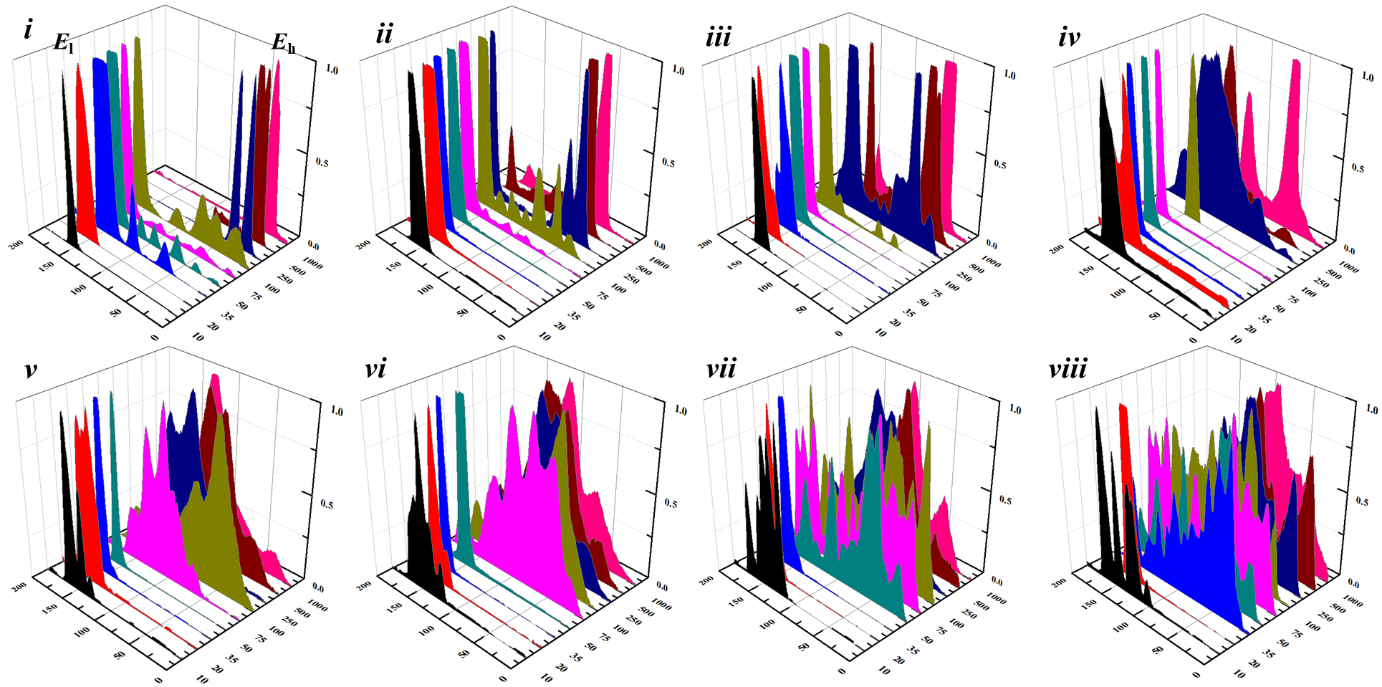


Figure S1 Normalized fluorescent intensity profiles of the particle trajectory patterns in different concentration PEO solutions. The input flow rate was increased from 10 to 1000 µl/min. These plots are able to show the particle ultimate distribution along the channel width after the migration. *i* Profiles of 0 ppm PEO solutions (DI water); *ii* Profiles of 5 ppm PEO solutions; *iii* Profiles of 10 ppm PEO solutions; *iv* Profiles of 25 ppm PEO solutions; *v* Profiles of 50 ppm PEO solutions; *vi* Profiles of 100 ppm PEO solutions; v*ii* Profiles of 250 ppm PEO solutions; v*iii* Profiles of 500 ppm PEO solutions.


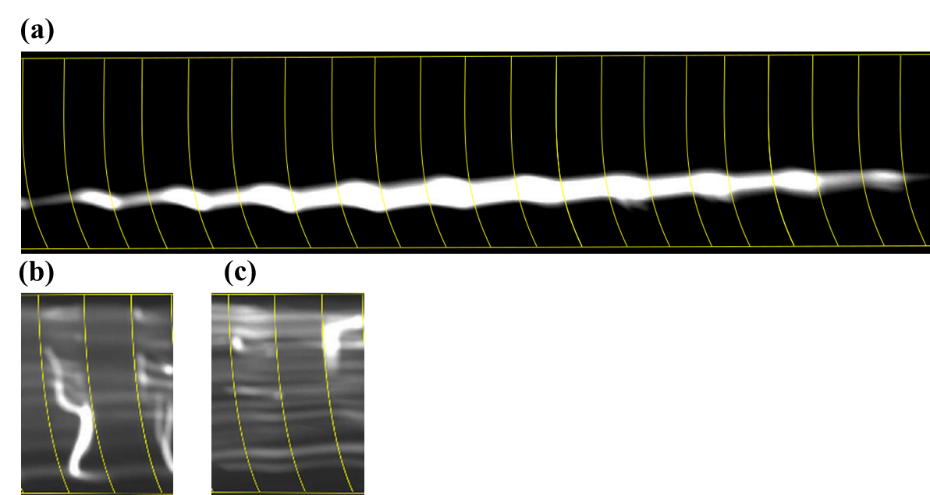


Figure S2 (a) Particle trajectory pattern in 25 ppm PEO solutions at the flow rate of 100 µl/min; (b) Particle trajectory pattern in 100 ppm PEO solutions at 60 µl/min; (c) Particle trajectory pattern in 250 ppm PEO solutions at 40 µl/min.
